# Bidirectional Hydrogen Electrocatalysis on Epitaxial Graphene

**DOI:** 10.1021/acsomega.2c00655

**Published:** 2022-04-04

**Authors:** Mikhail Vagin, Ivan G. Ivanov, Rositsa Yakimova, Ivan Shtepliuk

**Affiliations:** † Laboratory of Organic Electronics, Department of Science and Technology (ITN), Linköping University, SE-60174 Norrköping, Sweden; ‡ Semiconductor Materials, Department of Physics, Chemistry and Biology (IFM), Linköping University, SE-58183 Linköping, Sweden

## Abstract

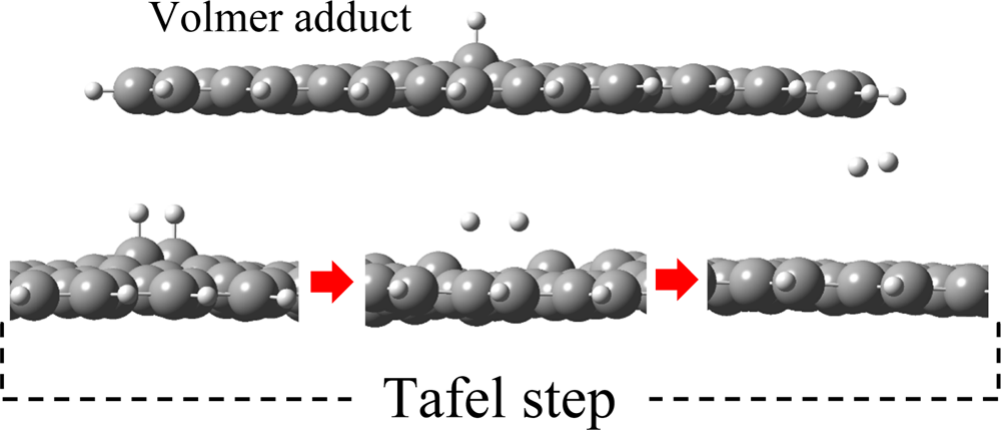

The climate change due to human activities stimulates the research on new energy resources. Hydrogen has attracted interest as a green carrier of high energy density. The sustainable production of hydrogen is achievable only by water electrolysis based on the hydrogen evolution reaction (HER). Graphitic materials are widely utilized in this technology in the role of conductive catalyst supports. Herein, by performing dynamic and steady-state electrochemical measurements in acidic and alkaline media, we investigated the bidirectional electrocatalysis of the HER and hydrogen oxidation reaction (HOR) on metal- and defect-free epigraphene (EG) grown on 4H silicon carbide (4H-SiC) as a ground level of structural organization of general graphitic materials. The absence of any signal degradation illustrates the high stability of EG. The experimental and theoretical investigations yield the coherent conclusion on the dominant HER pathway following the Volmer–Tafel mechanism. We ascribe the observed reactivity of EG to its interaction with the underlying SiC substrate that induces strain and electronic doping. The computed high activation energy for breaking the O–H bond is linked to the high negative overpotential of the HER. The estimated exchange current of HER/HOR on EG can be used in the evaluation of complex electrocatalytic systems based on graphite as a conducing support.

## Introduction

The very recent UN alerting report (from 2021-08-09)^1^ on the global climate change is uncompromising in its conclusion that the observable climate change is anthropogenic in origin. In this perspective, the implementation of clean technologies, such as the direct interconversion of chemical energy of water, an abundant and life-crucial resource of the Earth, and the electrical energy obtainable from renewable sources such as solar and wind generation, are prioritized.

Hydrogen is an environmentally friendly energy carrier of high density considered as a substituent of fossil fuels. However, hydrogen is produced nowadays by steam methane reforming [CH_4_(g) + H_2_O(g) → CO(g) + 3H_2_(g)] and coal gasification,^2^ which are not sustainable processes due to significant CO_2_ emission. Hydrogen production through water electrolysis, the electrochemical splitting of water into oxygen and hydrogen gases [2H_2_O(l) → 2H_2_(g) + O_2_(g)], is one of the key processes in the implementation of green hydrogen technologies because the utilized electricity can be obtained from renewable sources such as wind and solar energy. The inherent losses of applied electrical energy due to the slow kinetics of the hydrogen evolution reaction (HER) and the auxiliary reaction of oxygen evolution motivate the usage of electrocatalysts dispersed with a conductive matrix of graphitic materials.

Graphene is the simplest building block of graphitic materials. Due to the high anisotropy between in-plane and out-of-plane conduction originating from the planar conjugation of the sp^2^ bonds in graphene, the electron transfer rates observed at the edges are many orders of magnitude higher than those on the basal plane of graphene.^3,4^ Poor electrochemical activity of basal-plane free-standing monolayer graphene electrodes toward the HER is also attributed to its instability and loss of structural integrity during electrochemical measurements, which is particularly crucial for graphene grown on foreign substrates such as commonly used SiO_2_/Si wafers.^5^ The electrochemical activity of graphene developed *via* chemical vapor deposition is defined by the graphitic multilayered islands.^6^ The only way to revitalize graphene for electrochemical measurements is to use stable atomically flat large-area graphene sheets on native substrates. Such types of graphene can be exclusively formed through strictly controlled thermal decomposition of SiC wafers in an inert atmosphere,^7^ yielding high-quality monolayer epitaxial graphene (epigraphene/EG) with controllable exposure of a defect-free basal plane.^8^

In this work, we explored the kinetics of bidirectional HER/HOR electrocatalysis on EG grown on 4H-SiC by performing electrochemical measurements and first-principles theoretical calculations. The Volmer–Tafel mechanism of the HER on EG was computed and confirmed in the experiments. The reactivity of EG is assigned to the strain and doping effects originating from the interaction with the underlying SiC substrate. The estimated exchange current of the HER/HOR on EG can be used as a background in the evaluation of graphite-dispersed electrocatalytic systems.

## Experimental Section

An Autolab type III potentiostat (Autolab, EcoChemie, Utrecht, The Netherlands) was exploited for electrochemical measurements. An Ag/AgCl electrode in 3 M KCl and a platinum wire were employed as the reference and counter electrodes, respectively, for all measurements. All chemicals were purchased from Sigma (Sweden). Electrochemical measurements were performed using Milli-Q water from a Millipore Milli-Q system. The EG/SiC was used as a working electrode in the open electrochemical cell obtained from Redoxme AB. A monolayer EG electrode was synthesized through the Si sublimation process of 4H-SiC (0001) in an inductively heated graphite enclosure under controlled gas pressure–temperature–time conditions.^9^ As was confirmed by Raman mapping analysis and optical reflectance mapping (not shown here), the optimized growth conditions enabled the formation of high-quality graphene layers with high thickness uniformity (more than 90% of the substrate area is covered with monolayer graphene, and the rest is bilayer patches) and negligibly small defect density.

A micro-Raman setup based on a monochromator (Jobin-Yvon, model HR460) equipped with a couple-charged device (CCD) camera was used to investigate the structural quality of EG on 4H-SiC. The excitation source was a diode-pumped solid-state laser. The laser wavelength was 532 nm, while the laser power was 1 mW. The Raman spectra were obtained using a large-numerical-aperture (NA = 0.95) 100× micro-objective lens. The spectral resolution of the system was ∼5.5 cm^–1^.

## Theory

The HER at the EG on Si-face 4H-SiC (EG/SiC) was investigated based on hybrid gas-phase and solvated density functional theory (DFT) calculations performed by using the Gaussian 16 Rev. B.01 program package.^10^ To mimic the EG/SiC electrode, we employed a cluster model composed of a 4 × 5 first graphene layer located above the 4 × 5 buffer layer on the 4 × 4 Si-face surface of hexagonal SiC. Such a structure suggests that 26% of the carbon atoms belonging to the buffer layer (interfacial layer between SiC and graphene) are covalently bonded to the SiC surface, which agrees well with the experimental observations.^11^ All unsaturated carbon bonds were passivated by forming C–H bonds. The adsorption of hydrogen was simulated by full geometrical optimization of the H species located on EG with a self-consistent field convergence criterion of 10^–8^ and without symmetry restrictions. All calculations were carried out using the two-level ONIOM method implemented in Gaussian 16.^12^ The whole investigated system was divided into two regions: quantum mechanical (QM) and molecular mechanical (MM) regions. The QM region consists of the graphene top layer, hydrogen species, or water molecules, while the MM region comprises all atoms belonging to the SiC layers and buffer layer. To treat the QM region, we employed the hybrid dispersion-corrected DFT functional M06-2X.^13^ The 6-31G basis set for carbon, silicon, oxygen, and hydrogen atoms was used. Thus, the HER on EG/SiC was studied at the ONIOM (M06-2X/6-31G: UFF) level of theory. To investigate the role of the solvent, some calculations were conducted in the presence of water by using the polarizable continuum model (PCM).^14^ To shed more light on the role of the underlying 4H-SiC substrate in hydrogen binding by graphene, DFT calculations within periodic boundary conditions using the SIESTA code^15^ were additionally performed.

The HER most likely proceeds *via* a two-step mechanism.^16^ First, an atomic hydrogen intermediate adsorbed on the electrocatalyst surface is formed by the first electron transfer, the so-called Volmer step, for acidic and alkaline media, respectively
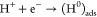

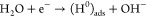
where (H^0^)_ads_ refers to the adsorbed hydrogen intermediate. Decisively, the second step of the HER proceeds either *via* exergonic recombination of adsorbates, the so-called Tafel step
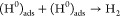
or *via* the second electron transfer, the so-called Heyrovsky step, for acidic and alkaline media, respectively
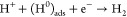

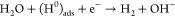


According to Nørskov *et al.*,^16^ the Gibbs free energy of H^0^ adsorption (Δ*G*_H^0^_) is the most meaningful descriptor of HER kinetics. Ideally, Δ*G*_H^0^_ should approach zero. However, in practice, it varies in a broad range of negative or positive values. The Δ*G*_H^0^_ value is estimated by the following equation
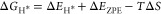
where Δ*E*_H*_ is the hydrogen adsorption energy, Δ*E*_ZPE_ is the difference in the zero-point energy (ZPE) between the adsorbed state and the gas phase, and Δ*S* is the entropy change of H^0^ adsorption. The Δ*E*_H^0^_, Δ*E*_ZPE_, and Δ*S* values can be calculated by using the following equations
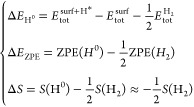
where , *E*_tol_^surf^, and  are the total energies of the surface with the adsorbed H^0^, adsorbate-free surface, and hydrogen molecule, respectively. The vibrational entropy for the adsorbed state is negligibly small and can be ruled out.

## Results and Discussion

The presence of high-quality monolayer EG on SiC is confirmed by Raman spectroscopy (Figure S1). Only characteristic Raman modes of EG (G, 2D, and 2D′) are present. No defect-related D peak is observed, suggesting the high crystallinity of graphene and low defect density. The Raman spectrum of EG also contains weak spectral features related to the buffer layer and G* peak related to intervalley scattering. The coverage of monolayer graphene on SiC determined by optical reflectance mapping^17^ is about 94.7% (Figure S2), testifying the assignment of observable electrochemical activity to monolayer graphene.

**Figure 1:**
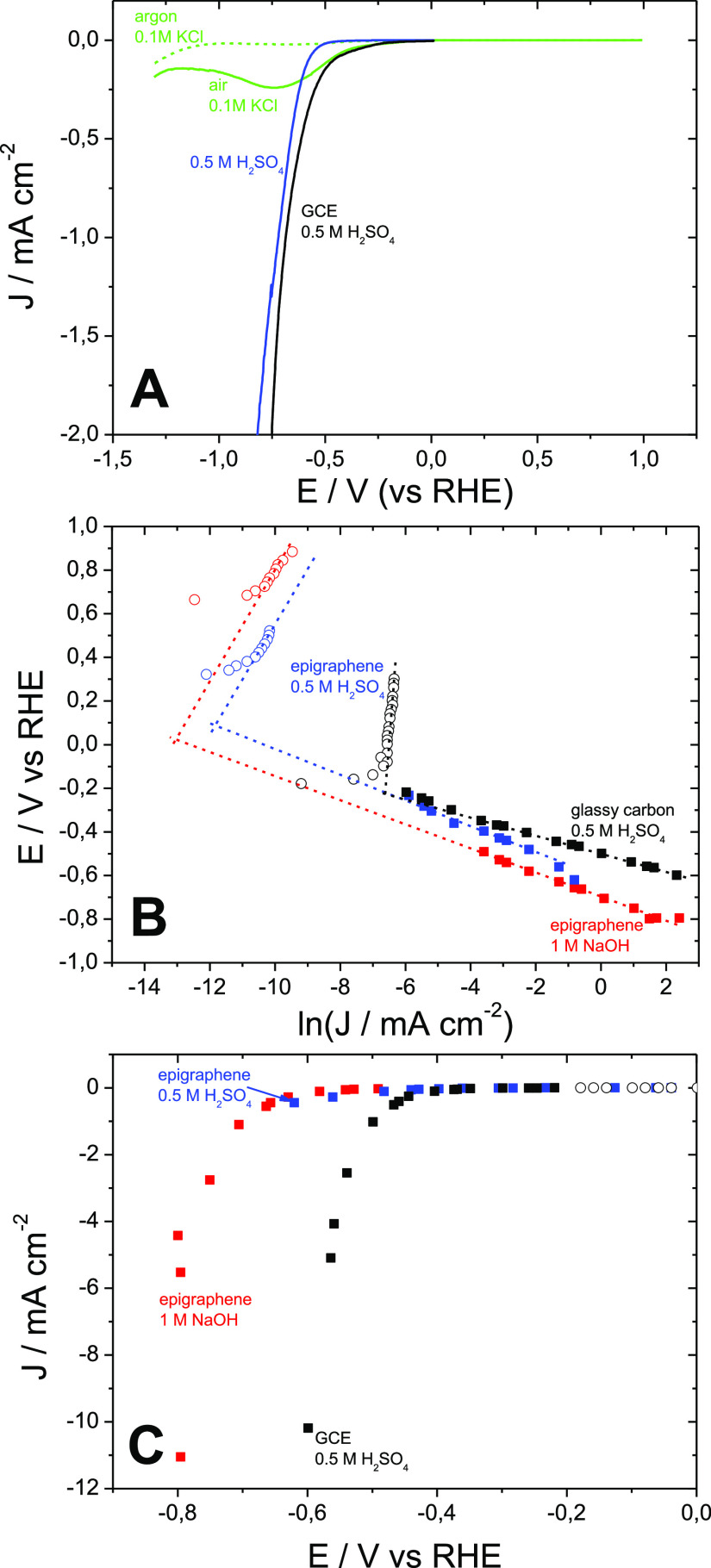
HER on EG. (A) Linear sweep voltammograms recorded on the EG monolayer [argon-saturated 0.5 M H_2_SO_4_ (blue) and air- and argon-saturated 0.1 M KCl (solid and dashed green curves, respectively)] and GCE (black curve, rotating-disk electrode, 900 rpm, argon-saturated 0.5 M H_2_SO_4_); IR-compensated steady-state polarization curves in Tafel and linear coordinates [(B,C), respectively] obtained on the EG monolayer (0.5 M H_2_SO_4_ and 1 M NaOH as red and blue symbols, respectively) and GCE (black symbols, rotating disk electrode, 900 rpm, argon-saturated 0.5 M H_2_SO_4_); oxidation and reduction currents are represented by open and filled symbols, respectively.

Both dynamic and steady-state electrochemical measurements were utilized for the quantification of HER kinetics on the EG monolayer in acidic and alkaline aqueous solutions. A glassy carbon electrode (GCE) was used as a reference material in the configuration of the rotating disk. Linear sweep voltammetry (LSV) showed the appearance of high negative currents at negative polarizations, manifesting the HER. Due to the open cell utilized in the measurements on EG, the background electrolyte was contaminated with oxygen, which led to the appearance of currents associated with the oxygen reduction reaction (ORR) (Figure 1A). This resulted in the overlap between the onset potential of the HER and ORR currents. The significant ohmic resistance implied that the EG electrode (*ca.* 1 kOhm) yielded the complex pattern of IR-compensated voltammograms (data not shown), which made the estimation of the kinetic parameters difficult using dynamic voltammetry data.

The use of the steady-state measurements (Figure 1B) allowed the observation of both negative and positive currents of the HER and HOR, respectively, manifesting bidirectional electrocatalysis on EG. The increase of the negative polarization led to the rise of HER-associated currents quantified by IR-compensated potentiometry. In contrast, the HOR-associated currents recorded by amperometry showed a minor dependence on positive polarization. This asymmetry is typical for the electrode HER/HOR and manifests the involvement of a slow surface chemical reaction. The low negative currents (up to 11 μA·cm^–2^, data not shown) were affected by the ORR, hindering the establishment of the HER/HOR equilibrium potential on the cell open to the air environment.

The recorded HER currents are free from the limitation by the reagent/product diffusion due to a fast virtual transport of proton *via* hydrogen bonds of water (Grotthuss mechanism). This implies that they are controlled by electron transfer only, the so-called kinetic currents, where direct Tafel analysis can be applied. The two distinctive mechanisms, namely, Volmer–Tafel and Volmer–Heyrovsky, can be distinguished by the measurements of the Tafel slope (reciprocal slope of potential *vs* logarithm of the current plot) of the polarization curve. Revealing the electrical energy loss due to the slow reaction kinetics, the Tafel slopes of the HER on EG estimated for more than 2 orders of magnitude of current density^18^ were 54 and 59 mV·decade^–1^ for alkaline (from 28 μA·cm^–2^ up to 11 mA·cm^–2^) and acidic electrolytes (from 2.8 μA·cm^–2^ up to 0.44 mA·cm^–2^), respectively. These are close to 60 mV·decade^–1^ assigned to the EC̅ mechanism,^18^ where the electron transfer step is followed by a rate-determining (slow) chemical step (C̅). In other words, the HER on the EG monolayer resembles the Volmer–Tafel mechanism. The minor change of the Tafel slope with pH (Figure 1B) illustrates the confinement of the mechanism. This is opposite to the Volmer–Heyrovsky pathway, which was found to dominate in the case of heteroatom-doped free-standing graphene (FSG) samples.^19^ Note that Tafel slopes close to 60 mV·decade^–1^ were reported for the bulk platinum disk electrode (at low overpotentials, 0.5 M H_2_SO_4_)^20^ and platinum (110) (at low overpotentials, 0.1 M KOH).^21^ Importantly, other carbon materials, being utilized mainly as HER catalyst supports, showed much higher values of kinetic loss estimated from the LSV measurements: graphite and heteroatom-doped graphene showed the Tafel slopes of 91–206 mV·decade^–1^ (in 0.5 M H_2_SO_4_) and 143–208 mV·decade^–1^ (in 0.1 M KOH).^22^ Here, we also report the Tafel slope of the HER on the GCE as 41 mV·decade^–1^ (from 4 μA·cm^–2^ up to 10 mA·cm^–2^; in 0.5 M H_2_SO_4_). This indicates that the electrode process proceeds *via* the Volmer–Heyrovsky mechanism, where the second electron transfer is the rate-determining step.^23^ Note that the Tafel slopes close to 40 mV/decade are also reported for platinum-based electrodes,^20^ inorganic platinum-free catalysts,^24^ and conducting polymers.^25^ The wide range of Tafel slopes observed for the HER on the carbonous materials suggests a significant effect of the state of the surface. The higher electronic density of states (DOS) at the edge plane in comparison with the basal plane of graphene as the simplest level of structural organization of all graphitic materials results in a few orders-of-magnitude higher rates of the electron transfer observed on edges determining chemical and electrochemical anisotropy.^3,8^ Bulk graphitic materials possess a large contribution of edges with high DOS, disabling the evaluation of electrochemical activity of the basal plane with low DOS, while the use of the EG monolayer enables these measurements.

Showing similar electrocatalytic activity, EG on SiC showed excellent stability during the radical-associated HER process in comparison with graphene developed on foreign substrates.^5^ This illustrates the importance of epitaxial growth, yielding the substrate-immobilized catalyst at the atomic scale.

**Figure 2:**
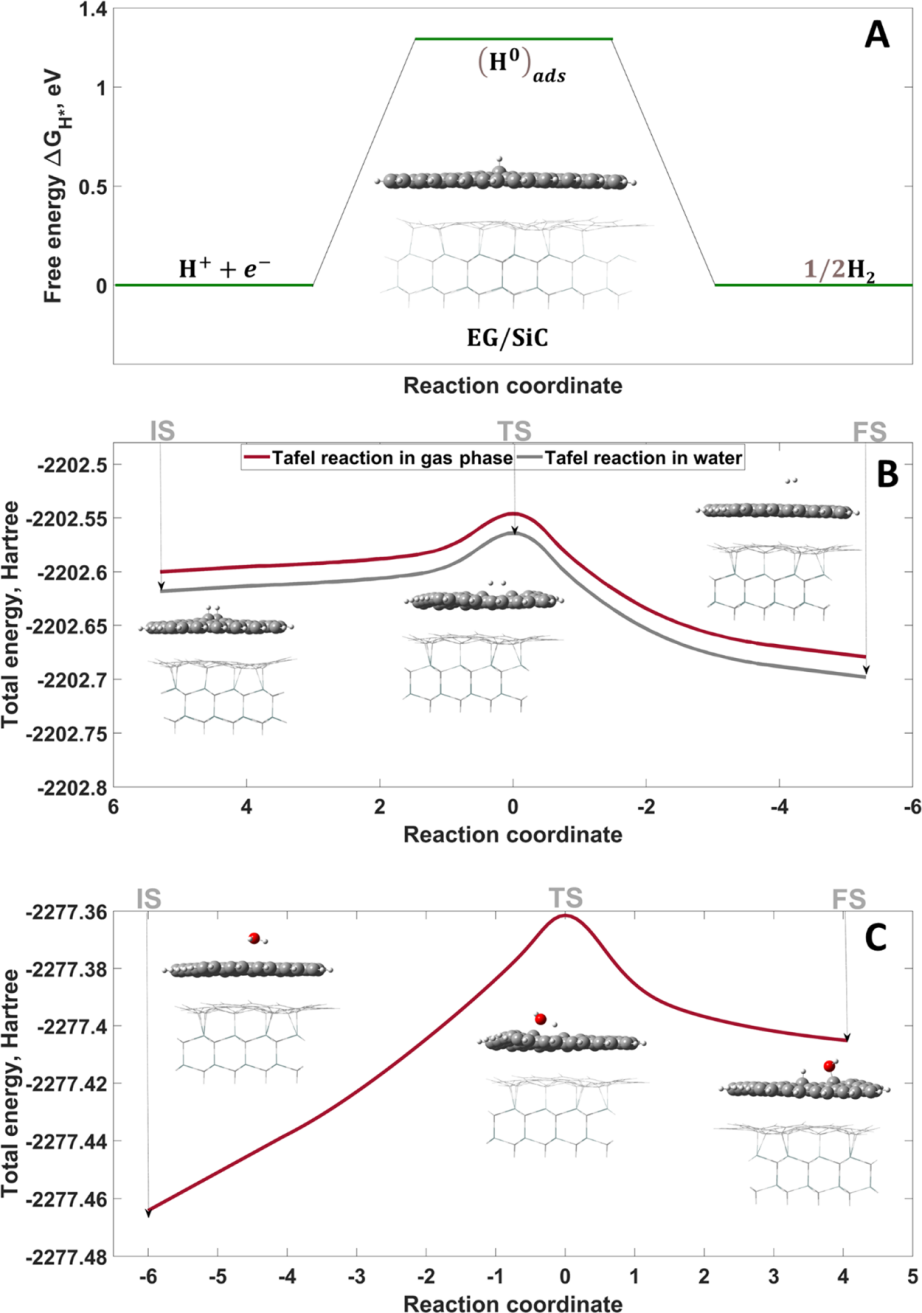
(A) DFT-computed reaction pathway on pristine EG on SiC. The image inside represents the relaxed structure of the adsorbed state; the energy barriers computed by calculation for the Tafel step and Heyrovsky step in vacuum and alkaline media [(B,C), respectively]. The relaxed structures inside correspond to the initial state (IS), transition state (TS), and final state (FS).

We computed the HER mechanism by DFT calculations. By placing one hydrogen proton above the EG surface, we initially studied the first Volmer step (inset in Figure 2A). Notably, certain atomic protrusions were found in the graphene top layer due to hydrogen adsorption. Hydrogen tends to be adsorbed at the top site of EG, and as a result, the carbon atom is lifted from the graphene plane. According to gas-phase calculations, Δ*G*_H^0^_ is equal to 1.243 eV (Figure 2A), which is lower than that for free-standing undoped graphene with a Δ*G*_H^0^_ value of 1.568 eV, while consideration of the solvent (water) effect gives larger values of 1.544 and 1.574 eV for FSG and EG/SiC, respectively (Table 1S). The above results mean that the hydrogen adsorption at FSG is an energetically unfavorable process, motivating the additional overpotential to drive the HER.^26^ It can be, however, assumed that the interaction between the topmost graphene layer with the underlying SiC support followed by charge transfer and strain appearance could reduce the Δ*G*_H^0^_ values to enhance the H^0^ adsorption, which makes the HER more favorable (less endothermic). Indeed, coupling of graphene with the substrate (e.g., γ-MoC) can induce new catalytic sites on graphene,^27^ thereby activating the nominally inert isolated graphene and improving its reactivity. Back to our case, DFT calculations using periodic boundary conditions (PBCs) (Figures S5 and S6) additionally corroborate the overall idea of improvement of the HER performance of graphene by means of the 4H-SiC substrate. More specifically, our results show that the average C–C bond length in graphene increases from 1.4232 to 1.5650 Å when graphene is placed on the SiC substrate. This is because the graphene layer experiences 9% tensile strain due to the lattice parameter mismatch between graphene and 4H-SiC. Furthermore, the SiC effect is also manifested in interfacial charge transfer. We notice that the topmost graphene layer composed of 32 carbon atoms accepts 0.31*e*^–^ (0.30 *e*^–^) electrons from 4H-SiC, as was predicted by Voronoi (Hirshfeld) charge population analysis. The electronic structure calculations (Figures S5–S7) confirm that, compared to FSG, EG on 4H-SiC is N-doped (the Dirac point is below the Fermi level). Also, from the analysis of the DOS, it is seen that the number of electronic states near the Fermi energy increases when moving from FSG and EG/SiC. Since HER performance strongly depends on the density of states near the Fermi energy level, it is reasonable to assume that this effect may additionally contribute to enhancement of the HER activity at EG/SiC. Kropp and Mavrikakis in their recent work^28^ proposed the mechanism underlying the HER on strained graphene. Particularly, it was revealed that tensile strain (C–C bond stretching) results in the weakening of the interaction between C p_z_ orbitals and, hence, an increase of the energy of occupied π states and decrease of the free energy of adsorption. Since EG models in both PBC and cluster limits imply that graphene experiences expansive strain, the above explains the enhanced catalytic activity of EG compared to FSG. However, it is important to emphasize that the real EG layers on 4H-SiC are usually compressively strained. Due to the thermal expansion coefficient mismatch between graphene and SiC,^29^ EG shrinks during the cooling process up to the formation of buckled ridges.^30^ Ridges are not observed however in a well-controlled cool-down process. This indicates the coexistence of spatially separated compressively and tensely strained regions of EG. Significantly, according to Kropp and Mavrikakis,^28^ the Volmer reaction for compressively strained graphene becomes kinetically more favorable even than that for expanded graphene. In this case, lower energy penalty needs to be paid to rehybridize orbitals upon hydrogen adsorption. Nevertheless, in the frames of the current work, we considered the HER only for tensely strained EG electrode.

The experimental results presented above suggest that the Volmer–Tafel route dominates over the Volmer–Heyrovsky one for the EG electrode. From a theoretical point of view, this means that the activation barriers of the Heyrovsky step are expected to be much higher than those of the competing Tafel step in both vacuum and water. As a direct consequence, newly incoming hydrogen protons will always tend to occupy the C top site of EG but not react with already adsorbed (H^0^)_ads_ to form molecules of hydrogen. Therefore, we will further focus on the Tafel step only (see animation files Videos S1 and S2 in the Supporting Information). We first positioned the second hydrogen proton in the proximity of the C top site of graphene to make sure that the second H^0^ would form. The resulting distance between the adsorbed hydrogen species was found to be 2.89 Å in the gas-phase and aqueous conditions. Next, we performed additional DFT frequency and intrinsic reaction coordinate (IRC) calculations to find the transition state for the Tafel reaction (Figure 2B). The transition-state structures were confirmed by the presence of only one imaginary frequency at −1921.17 and −1932.82 cm^–1^ in the gas phase and in water, respectively. The activation barriers were 28.74 and 29.03 kcal mol^–1^, respectively (Table 2S). In Table 2S, it can be also seen that the estimated barriers are smaller than the reaction barriers for the Tafel step for FSG. It becomes obvious that the SiC substrate plays an important role in lowering the activation energy barrier for the Tafel step in EG.

Back to the experimental results, the exchange current densities of the HOR/HER equilibrium on EG as a driving force-free rate of the process estimated from the intercept of the extrapolated dependencies of the HER and HOR currents from the Tafel regions showed the values of 7.9 × 10^–9^ and 2.0 × 10^–9^ A cm^–2^ for acidic and alkaline electrolytes, respectively. This locates the rates of HER on EG below coinage metals (gold: 1.3 × 10^–6^ A cm^–2^ and 0.6 × 10^–6^ mkA cm^–2^ for acidic^31^ and alkaline^32^ electrolytes, respectively; silver: 1.3 × 10^–8^ and 5.0 × 10^–8^ A cm^–2^ for acidic^31^ and alkaline^32^ electrolytes, respectively) and above sp-metals [bismuth: 1.0 × 10^–11^ A·cm^–2^ and 1.6 × 10^–11^ A·cm^–2^; cadmium: 2.5 × 10^–12^ A·cm^–2^ and 5 × 10^–13^ A·cm^–2^; gallium 8.0 × 10^–11^ A·cm^–2^ and 1.2 × 10^–11^ A·cm^–2^; mercury: 3.2 × 10^–13^ A·cm^–2^ and 3.2 × 10^–14^ A·cm^–2^; lead: 5 × 10^–12^ A·cm^–2^ and 1 × 10^–11^ A·cm^–2^; and tin: 5.0 × 10^–10^ A·cm^–2^ and 1.0 × 10^–10^ A·cm^–2^ for acidic^31^ and alkaline^32^ electrolytes (for all sp-metals), respectively]. For comparison, the exchange current density for the HER/HOR equilibrium on the GCE was 1.3 × 10^–6^ A cm^–2^, which is more than 2 orders of magnitude larger than on EG. The experimental exchange current density for the HER on EG is within the region of theoretically estimated values (Supporting Information Note 1). With respect to the electrochemically active surface area (EASA) of the electrochemical interfaces, the capacitive current densities were more than 30 times smaller on the EG monolayer in comparison with the GCE. However, the normalization of the estimated exchange current densities on the capacitive current densities^33^ to account for the effect of the EASA on the process rate is invalid because the anisotropy of graphene yields different values of specific capacitance at the edge and basal planes represented on the bulk carbon material (GCE).

The onset potential of the HER on EG in the alkaline electrolyte estimated from the linear coordinate plot (Figure 1C) was *ca.* −0.65 V, which is 0.2 V more negative than that for the GCE (*ca.* −0.45 V) and much more than the reported values for high-performance HER catalysts,^34^ benchmarking the kinetic loss of the HER on graphitic materials as a support for the catalysts.

It was assumed that the HER in alkaline media proceeds *via* the Heyrovsky reaction as a rate-determining step.^31^ The computed adsorption energy of water molecules on EG (Δ*G*_H_2_O_) showed a negative value of −0.25 eV, illustrating the physisorption character of interaction. Physisorbed H_2_O accommodates electrons forming adsorbed intermediates (H^0^)_ads_ and OH^–^. Due to strong electrostatic affinity, OH^–^ is also immobilized by the surface of the EG, thereby blocking the active sites (so-called OH blocking). Two adsorbed H^0^ species on neighboring adsorption sites recombine with each other, forming a hydrogen molecule through the Tafel step, which is unaffected by the medium’s acidity. The energetics of the Heyrovsky step on EG in alkaline media (Figure 2C) is illustrated by the activation energy for breaking the O–H bond in water, which is 66.8 kcal mol^–1^. The transition-state structure was confirmed by the presence of only one imaginary frequency at −1631.35 cm^–1^. The low HER reactivity of EG in alkaline media illustrated by the high value of the activation energy might be a result of the presence of the additional step of water adsorption (pre-Volmer step)
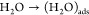
which happens before the fast electron transfer (Volmer step on the surface)
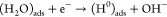


## Conclusions

The combination of defect-free monolayer EG with the SiC substrate enables investigation of the ground-level electrocatalysis for the atomically thin sheet of sp^2^ bonded carbon atoms. The results of dynamic and steady-state electrochemical measurements complemented by the DFT data indicate the Volmer–Tafel mechanism for the HER on EG. Unlike carbonaceous materials, EG showed a much lower Tafel slope (∼60 mV/decade), indicating that the initial hydrogen atom adsorption is followed by a rate-determining (slow) chemical step. The graphene interaction with the native substrate achieved *via* epitaxy yielded both ultimate stability in the conditions of aggressive radical-associated process and reactivity enhancement in comparison with the FSG or foreign substrate-deposited graphene. The estimated value of HER/HOR exchange current on EG can be used in the evaluation of kinetics of complex electrocatalytic systems based on graphitic dispersions.

## Supporting Information Available

Mean Raman spectrum and the reflectance map of monolayer EG, optimized geometries and flat band structures of FSG and EG before and after hydrogen adsorption, charge redistribution on EG, visualization of the Tafel step on EG in vacuum and in the solvent, and estimation of the exchange current densities from the computed values of Δ*G*_H^0^_ (PDF)
