# Supplementary material for: Bidirectional Hydrogen Electrocatalysis on Epitaxial Graphene
Source: ACS Omega. 2022 Apr 4;7(15):13221–7. doi: 10.1021/acsomega.2c00655 (PMC9025984; doi:10.1021/acsomega.2c00655)
Supplement: Supplementary file 1 — ao2c00655_si_001.pdf [file ao2c00655_si_001.pdf]

SUPPORTING INFORMATION

Bidirectional hydrogen electrocatalysis on epitaxial graphene

Mikhail Vagin[1]*, Ivan G. Ivanov[2], Rositsa Yakimova[2], Ivan Shtepliuk[2]*

[1]Laboratory of Organic Electronics, Department of Science and Technology (ITN), Linköping University, SE-60174 Norrköping, Sweden

[2]Semiconductor Materials, Department of Physics, Chemistry and Biology (IFM), Linköping University, SE-58183 Linköping, Sweden

*Corresponding author:

mikhail.vagin@liu.se

ivan.shtepliuk@liu.se

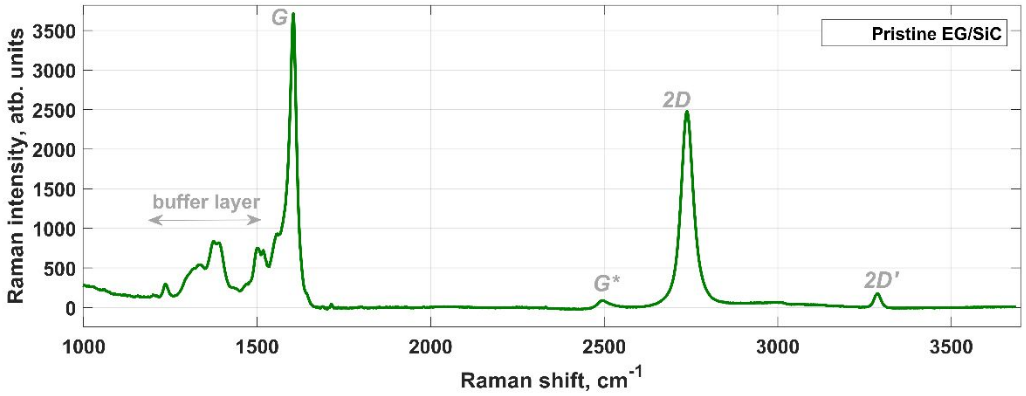

Figure S1. The mean Raman spectrum of monolayer epitaxial graphene after subtracting the contribution from the SiC substrate.

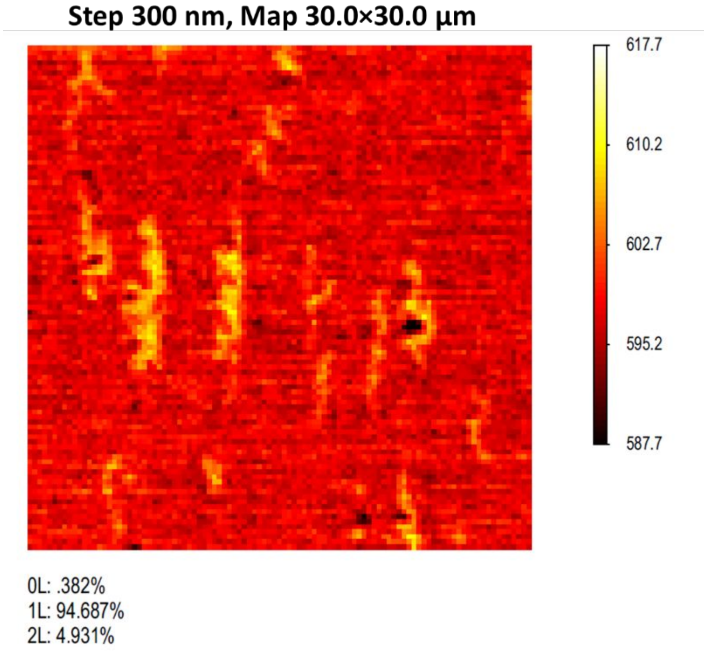

Figure S2. The reflectance map on a 30×30 μm^2 area displaying the graphene thickness uniformity. Monolayer epitaxial graphene (red) with patches of bilayer epitaxial graphene (yellow) and buffer layer (black) are distinguishable.

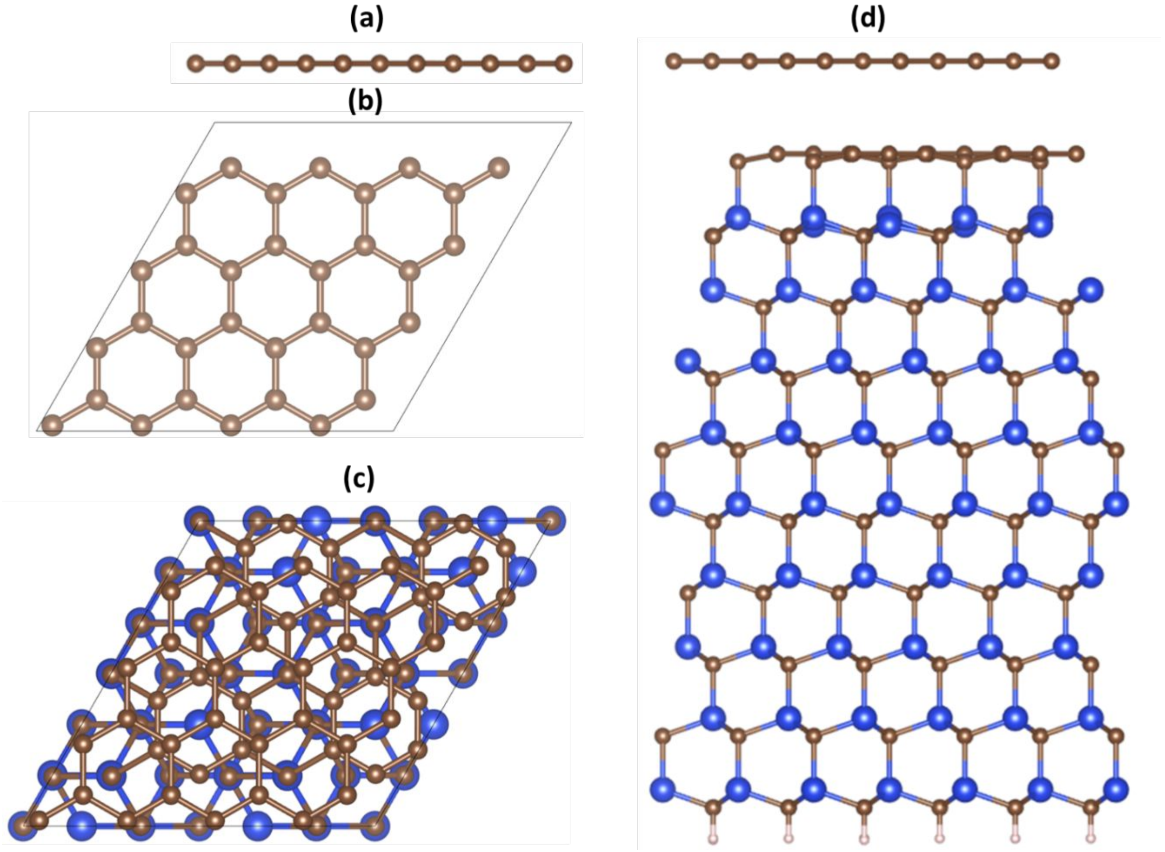

Figure S3. Optimized geometries of FSG (a – side view, b – top view) and EG/SiC (c – top view, c – side view) electrodes using PBC model. Brown, blue, and whitish balls designate carbon, silicon and hydrogen atoms, respectively.

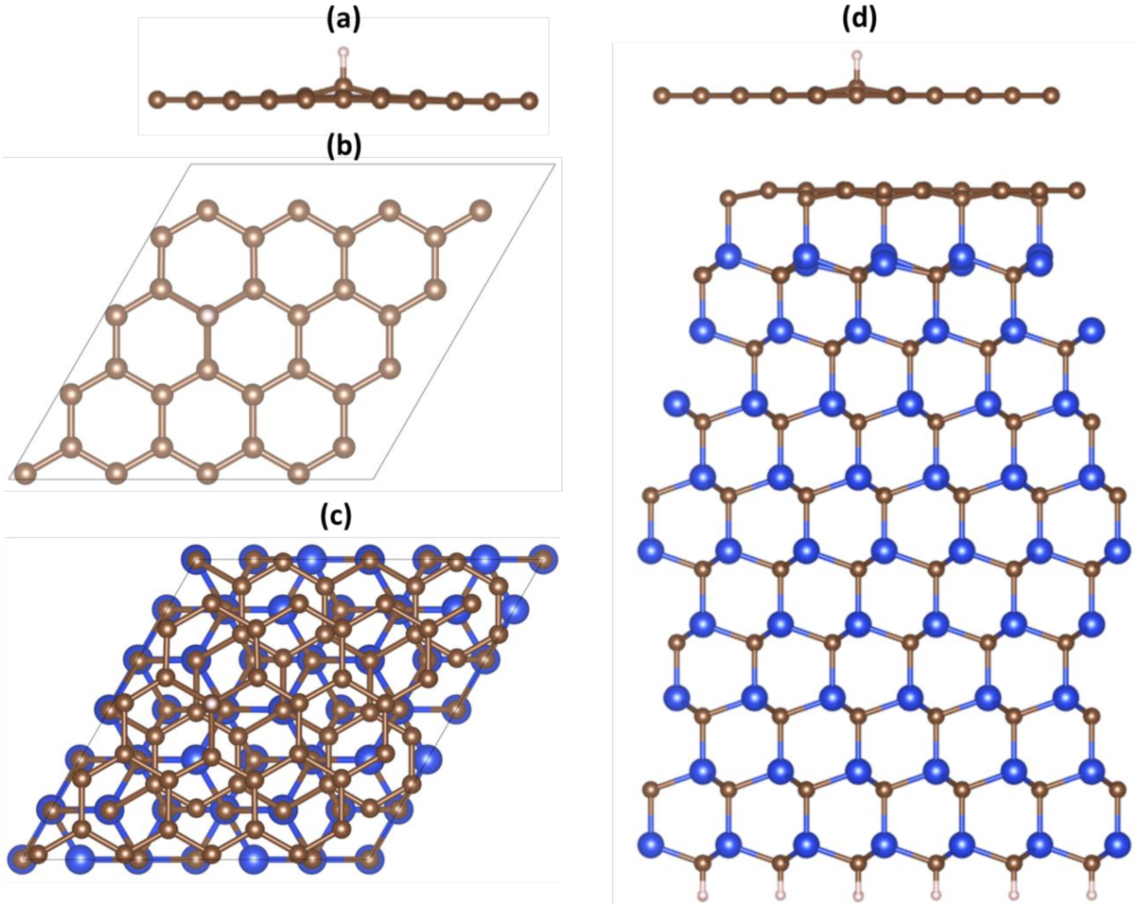

Figure S4. Optimized geometries of FSG (a – side view, b – top view) and EG/SiC (c – top view, c – side view) electrodes after hydrogen adsorption using PBC model. Brown, blue, and whitish balls designate carbon, silicon and hydrogen atoms, respectively.

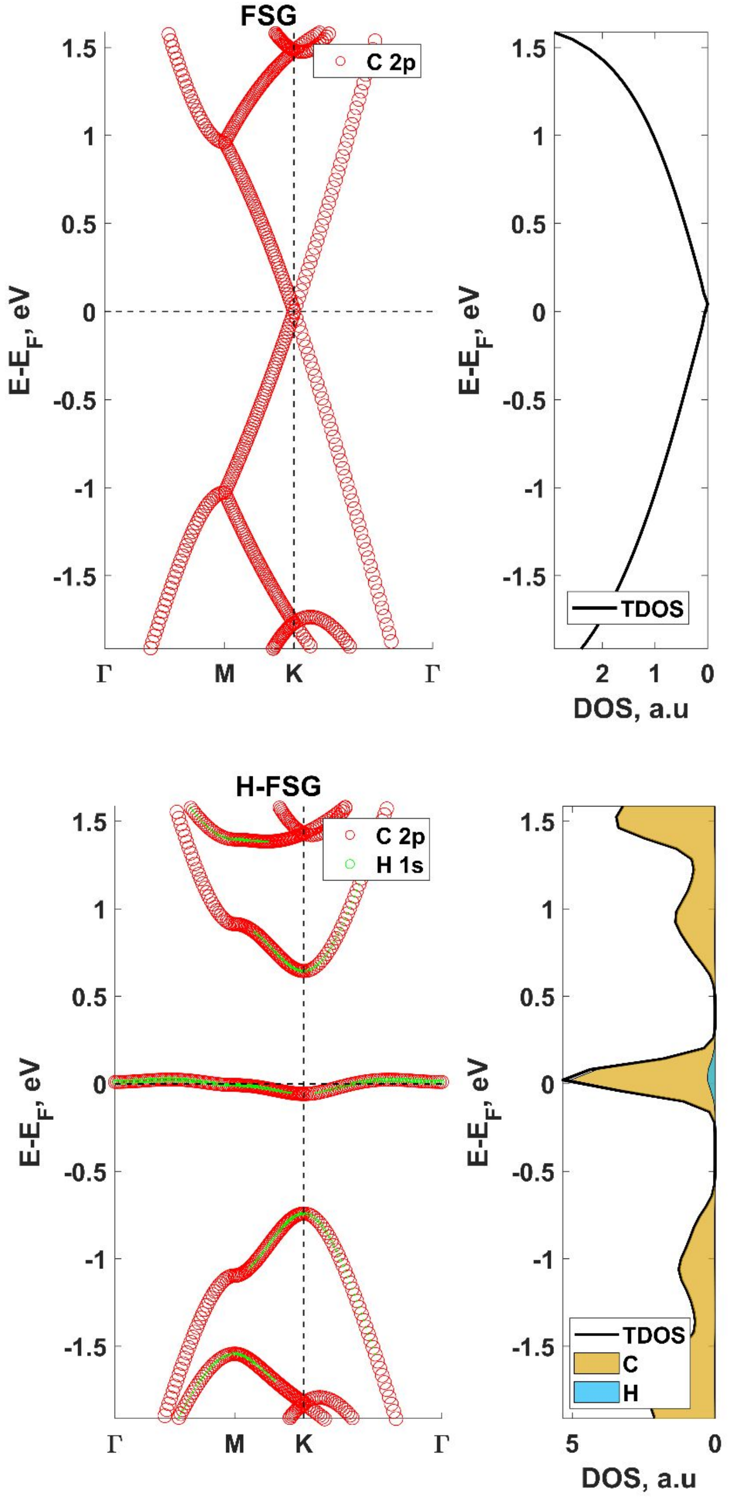

Figure S5. Fat band structures of free-standing graphene before (top panel) and after (bottom panel) H adsorption. Contributions of C 2p and H 1s orbitals to the bands are demonstrated. The size of each circle is proportional to the corresponding orbital contribution to each band. The Fermi level was set to 0. eV. The panels from the right show corresponding total density of states (DOS) and projected density of states (PDOS). The band-structure and DOS/PDOS calculations were performed using 9 × 9 × 1 and 60 × 60 × 1 *k*-point Monkhorst-Pack meshes at GGA-PBE/DZP level of DFT by SIESTA code, respectively.

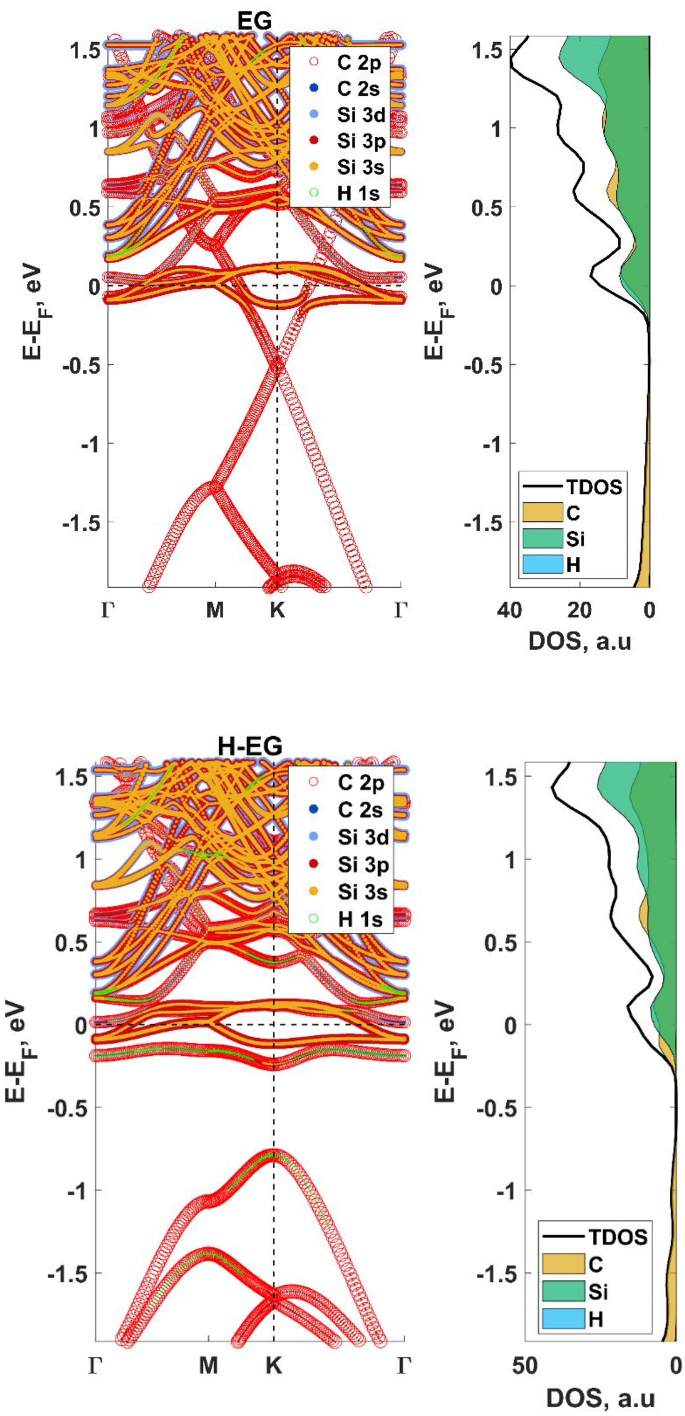

Figure S6. Fat band structures of epitaxial graphene on 4H-SiC before (top panel) and after (bottom panel) H adsorption. Contributions of key orbitals to the bands are demonstrated. The size of each circle is proportional to the corresponding orbital contribution to each band. The Fermi level was set to 0. eV. The panels from the right show corresponding total density of states (DOS) and projected density of states (PDOS). The band-structure and DOS/PDOS calculations were performed using 9 × 9 × 1 and 60 × 60 × 1 *k*-point Monkhorst-Pack meshes at GGA-PBE/DZP level of DFT by SIESTA code, respectively.

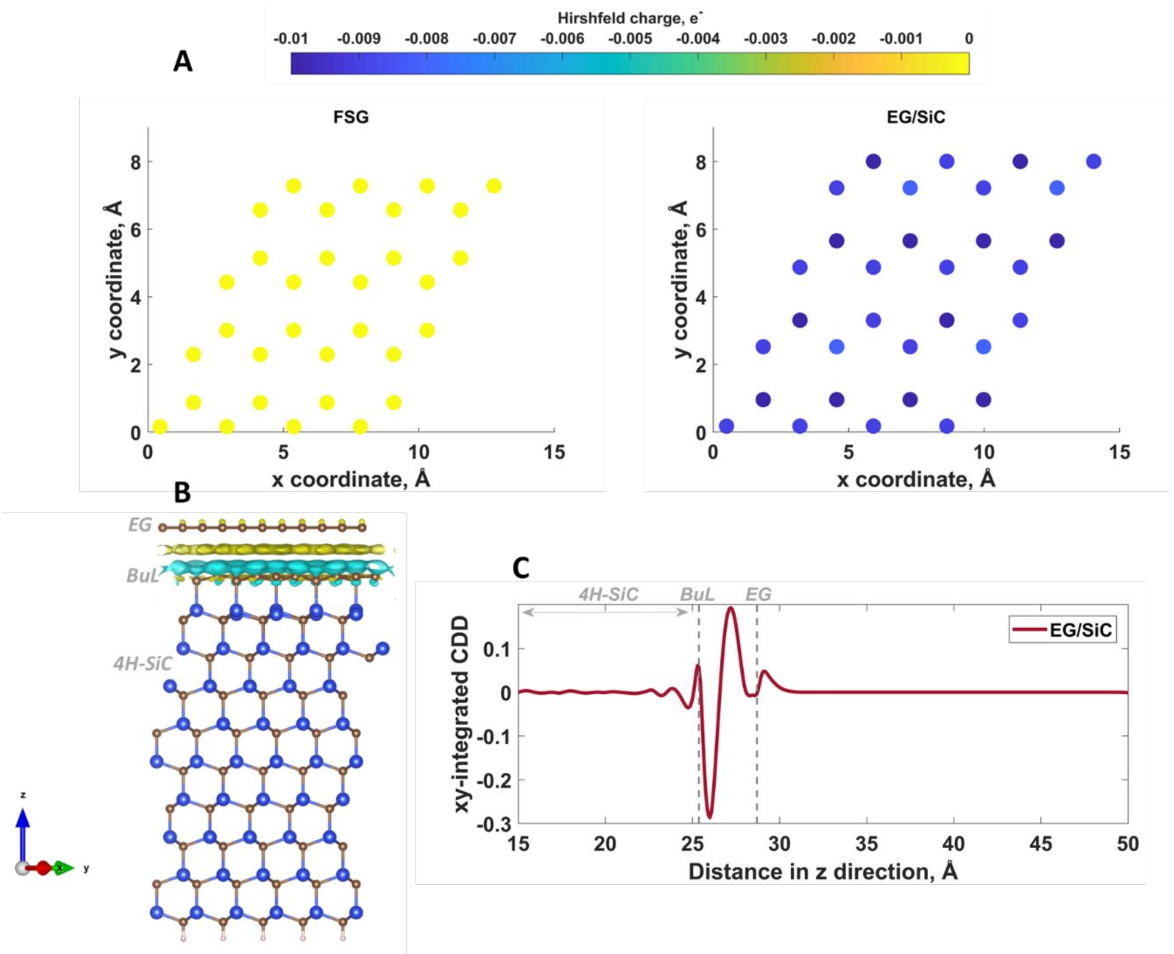

Figure S7. (A) Charge redistribution maps (made based on Hirshfeld population analysis) for free-standing graphene and epitaxial graphene on 4H-SiC. (B) The 3D CDD plot with an iso-surface value of 0.0005 e·Å^{-3} for EG/SiC. Yellow and cyan colors represent accumulation and depletion of charges, respectively. (C) The planar-averaged charge density difference for the EG/SiC vs. *z*-coordinate. The vertical lines designate the location of the buffer layer and epitaxial graphene, respectively. Positive and negative values of the CDD represent charge accumulation and charge depletion regions, respectively. The calculations of charge properties were carried out using 16 × 16 × 1 *k*-point Monkhorst-Pack mesh at GGA-PBE/DZP level of DFT by SIESTA code. The charge density difference was calculated by using following equation: $\Delta\rho = \rho_{EG/SiC} - \rho_{SiC} - \rho_{EG}$, where $\rho_{EG/SiC}$ is the electronic density of the interacting EG/SiC system, while ρ_{SiC} and ρ_{EG} are electron densities of the isolated SiC and EG, respectively.

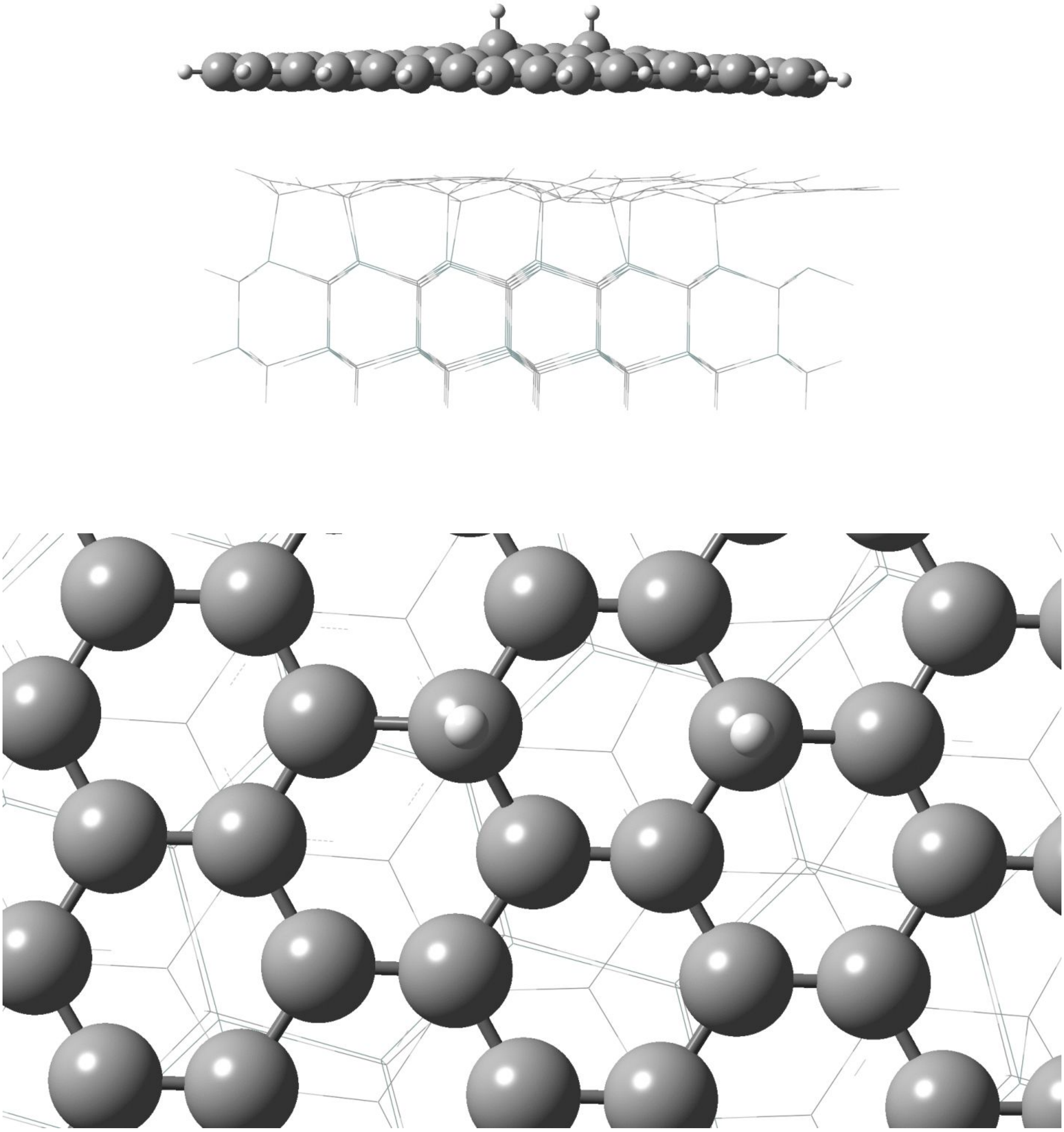

Video S1. Visualization of Tafel reaction at EG/SiC electrode in vacuum conditions. Top and side views of EG/SiC electrode are demonstrated. All calculations were performed using cluster model. Grey and white balls correspond to carbon and hydrogen atoms, respectively. While grey wireframe designates SiC substrate covered with buffer layer.

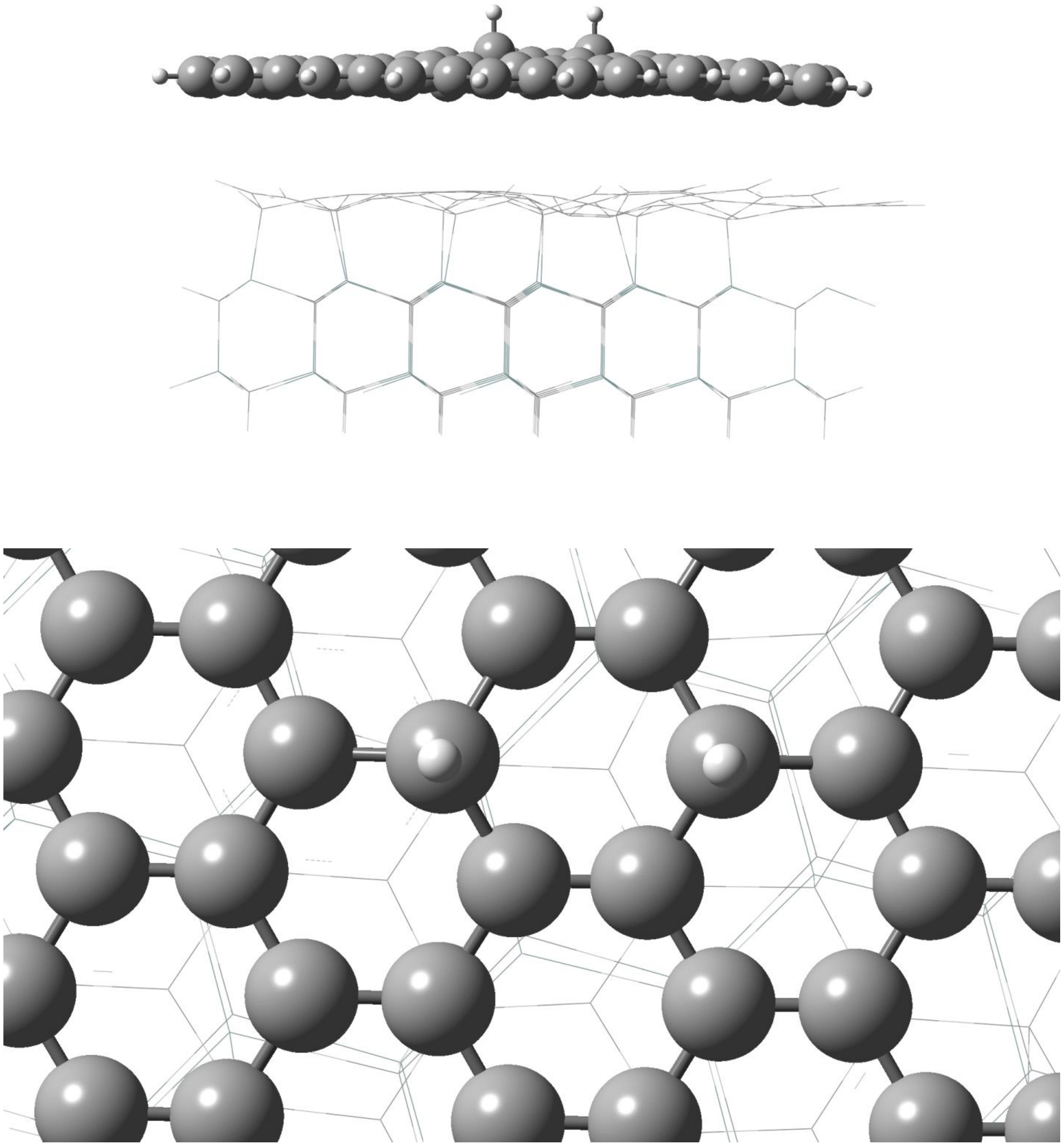

Video S2. Visualization of Tafel reaction at EG/SiC electrode immersed in solvent. Top and side views of EG/SiC electrode are demonstrated. All calculations were performed using cluster model. Grey and white balls correspond to carbon and hydrogen atoms, respectively. While grey wireframe designates SiC substrate covered with buffer layer.

Supporting Note 1

The turnover frequency (TOF) can be calculated from computed value of ΔG_{H^0}using an equation from transition state theory [DOI: 10.1021/jacs.5b03329]:

$$TOF = (k_B T/h) \times \exp\left(-\Delta G_H 0/RT\right) \quad (1)$$

where k_B is the Boltzmann constant, h is the Planck constant, R is the gas constant, T is the temperature. The theoretical value of exchange current density can be estimated by using TOF and number of active sites per surface area (*N*) [DOI: 10.1021/acs.nanolett.8b01335]:

$$j = 2qN \times TOF \quad (2)$$

where q is elementary charge. *N* was estimated by dividing a number of carbon atoms in graphene slab (32 for PBC model and 58 for cluster model, respectively) by surface area of epitaxial graphene (400.04 Å^2 and 613.18 Å^2**,** respectively). The numbers of active sites per surface area were $7.9 \cdot 10^{14}$ sites/cm^2 and $9.4 \cdot 10^{14}$ sites/cm^2 for PBC model and for cluster model, respectively.

The estimated values of the exchange current densities were 4.82×10^{-6} A·cm^2 and 2.71×10^{-12} A·cm^2 for PBC and cluster models, respectively. The experimental exchange current density for HER on epitaxial graphene is located within the region of theoretically-estimated values.

Table 1S. The calculated parameters describing the Volmer step ($H^+ + e^- \rightarrow (H^0)_{ads}$) in vacuum and acidic media at the free-standing graphene and epigraphene. The data in square brackets correspond to the values adsorption energy and free energy of adsorption extracted from SIESTA calculations using PBC conditions.

Phase	Substrate	Adsorption energy, eV	Zero-point energy, eV	Free energy of adsorption, eV
Gas	FSG	1.1854 [1.5015]	0.3229 [0.32]	1.5685 [1.8215]
	EG/SiC	0.86 [0.5438]	0.32 [0.32]	1.24 [0.8638]
Water	FSG	1.1905	0.3236	1.5744
	EG/SiC	1.16	0.32	1.54

Table 2S. The computed parameters of the Tafel step ($(H^0)_{ads} + (H^0)_{ads} \rightarrow H_2$) in different phases on non-neighboring C top sites of epitaxial graphene. The data for free-standing graphene are also provided.

Phase	Substrate	Imaginary frequency for transition state, cm^{-1}	Activation energy, kcal $mole^{-1}$	Rate of reaction, s^{-1}
Gas	FSG	-1844.80	33.0296	$3.8011 \cdot 10^{-12}$
	EG/SiC	-1921.17	28.7418	$5.2868 \cdot 10^{-9}$
Water	FSG	-1862.17	33.5768	$1.5093 \cdot 10^{-12}$
	EG/SiC	-1932.82	29.0374	$3.2102 \cdot 10^{-9}$
